# Le plasmocytome solitaire osseux: à propos de 3 cas et revue de la littérature

**DOI:** 10.11604/pamj.2016.25.219.10933

**Published:** 2016-12-06

**Authors:** Karim Masmoudi, Emna Elleuch, Rim Akrout, Mohamed Amine Mnejja, Afef Feki, Mariam Ezzeddine, Sofiène Baklouti, Ben Jemaâ

**Affiliations:** 1 Rhumatology Department, Hédi Chaker University Hospital, El-Aïn Street Km 0,5 zip code 3029 Sfax, Tunisia; 2 Infectious diseases Department, Hédi Chaker University Hospital, El-Aïn Street Km 0,5 zip code 3029 Sfax, Tunisia

**Keywords:** Plasmocytome solitaire, tumeur osseuse, myélome multiple, Solitary plasmocytoma, bone tumor, multiple myeloma

## Abstract

Les plasmocytomes solitaires sont rares et représentent moins de 5 % de l'ensemble des proliférations plasmocytaires. Son diagnostic repose sur la mise en évidence d'une tumeur localisée, constituée de cellules plasmocytaires monoclonales cytologiquement identiques à celles du myélome multiple en l'absence d'autres signes d'une forme disséminée. Le but de ce travail est d'effectuer une étude rétrospective de 3 observations de plasmocytomes solitaires osseux associée à une revue détaillée de la littérature décrivant les caractéristiques diagnostiques, thérapeutiques et évolutives de cette entité rare.

## Abstract (en)

Solitary plasmocytomas are rare, accounting for less than 5% of all plasmocytes proliferations. Its diagnosis is based on the presence of localized tumor of monoclonal plasma cells cytologically identical to those of multiple myeloma, in the absence of other signs of a disseminated form. This study aims to carry out a retrospective study of three cases of solitary bone plasmocytomas associated with a detailed review of the literature describing the diagnostic, therapeutic and evolving characteristics of this rare entity.

## Introduction

Le plasmocytome solitaire osseux (PSO) est une tumeur osseuse primitive rare caractérisée par la prolifération monoclonale de cellules plasmocytaires malignes localisées à un segment osseux, sans signe d'envahissement systémique [1–5]. La localisation vertébrale est la plus fréquente. Le PSO peut aussi atteindre les côtes, le sternum, l'os iliaque et les os longs [2]. Les auteurs de ce travail rapportent 3 observations de plasmocytomes solitaires osseux afin d'étudier leurs aspects diagnostiques, thérapeutiques et évolutifs.

## Patient et observation

**Figure 1:**
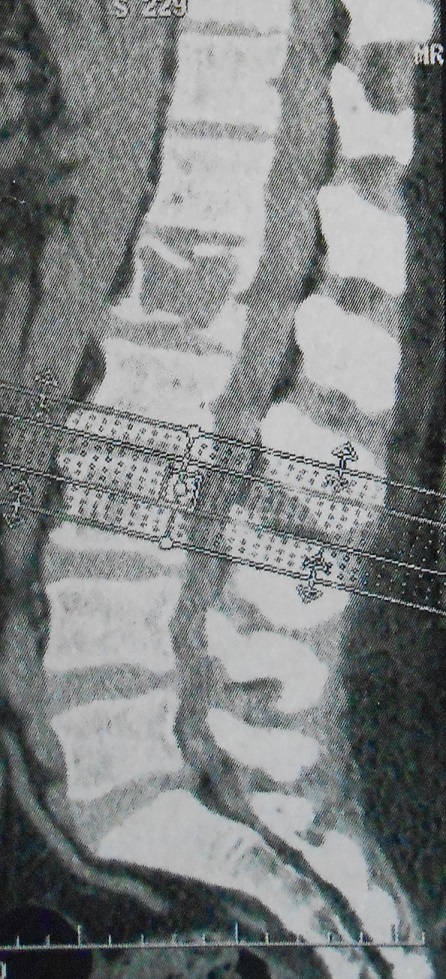
Reconstruction sagittale tomodensitométrique (TDM) du rachis lombaire montrant une fracture pathologique du corps vertébral de L1

**Figure 2:**
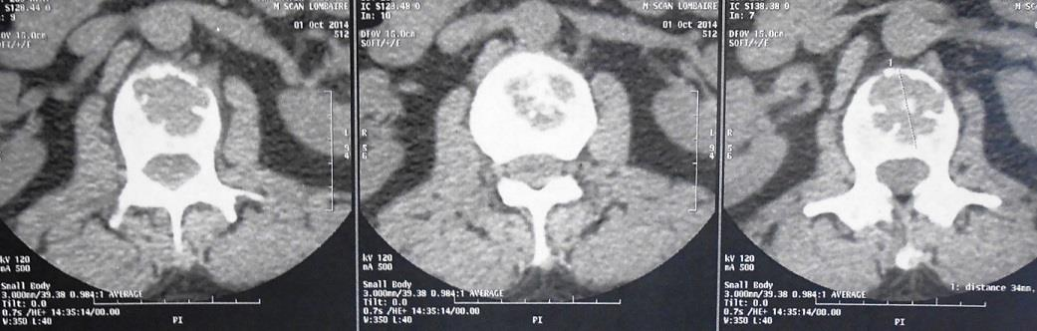
Coupes horizontales TDM passant par L1 montrant une lésion ostéolytique du corps vertébral sans atteinte du mur postérieur ni rétrécissement du canal vertebral

**Observation 1 :** un homme âgé de 47 ans sans antécédents pathologiques, consulte pour lombalgies chroniques d'allure mécanique, irradiant vers le membre inférieur droit, évoluant depuis 2 mois, sans signes d'atteinte neurologique. Les bilans inflammatoire et phosphocalcique étaient normaux, l'immuno-électrophorèse des protéines plasmatiques (IEPP) était sans particularités. Le patient a été exploré par une TDM rachidienne montrant une lésion tumorale du corps vertébral de L1 de densité tissulaire, qui se réhausse de façon hétérogène responsable d'un tassement modéré sans signe d'épidurite ou d'atteinte de l'arc postérieur (Figure 1, Figure 2). Le patient a eu une biopsie chirurgicale de la lésion dont l'examen histologique et immuno-histochimique avait conclu à un plasmocytome vertébral de phénotype IgG lambda. Une radiothérapie a été indiquée et appliquée, avec une évolution favorable au dernier recul de 12 mois sans aucun signe clinico-radiologique de récidive loco-régionale.

**Figure 3:**
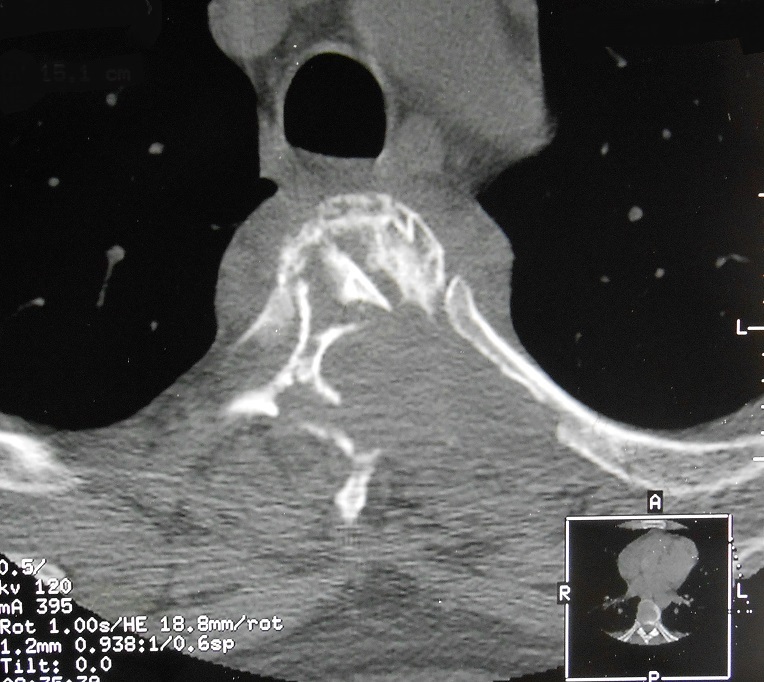
Coupe TDM horizontale passant par D3 montrant un éclatement du corps vertébral, associé à une ostéolyse de l’arc postérieur

**Figure 4:**
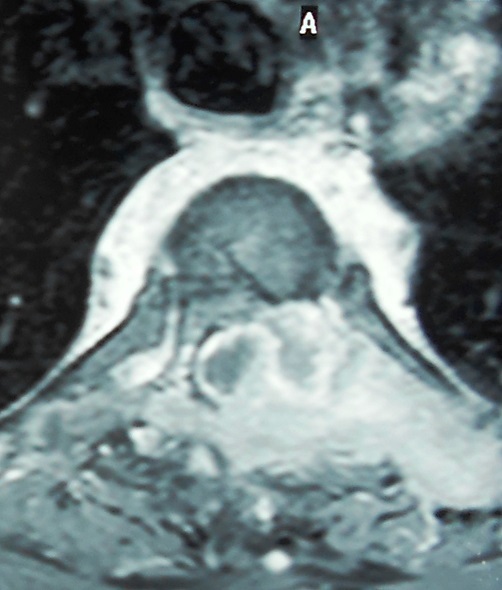
Coupe horizontale d’une IRM rachidienne passant par D3 montrant une ostéolyse de l’hémi-arc postérieur gauche associé à une compression et une latéro-déviation de la moelle dorsale

**Figure 5:**
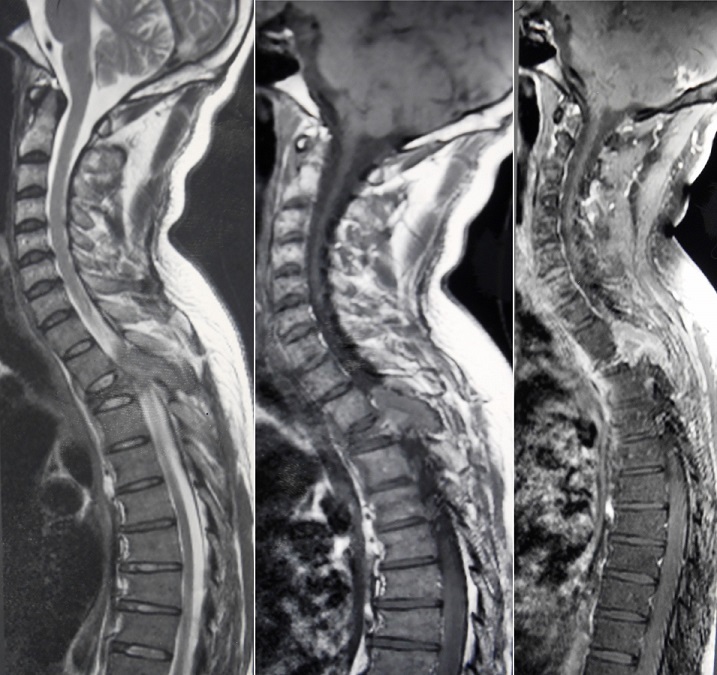
Coupes sagittales d’une IRM rachidienne (séquence T1, T2 avant et après saturation de la graisse) montrant une lésion ostéolytique de D3 en iso-signal T1, hyper-signal T2, avec une épidurite et une compression médullaire

**Figure 6:**
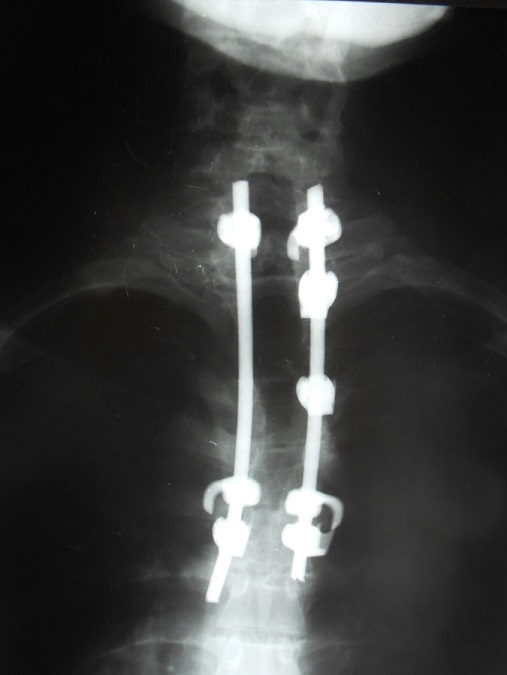
Radiographie post-opératoire après laminectomie décompressive et stabilisation du rachis dorsal par instrumentation postérieure

**Observation 2:** un homme âgé de 37 ans sans antécédents pathologiques, se plaint de dorsalgies d'allure inflammatoire évoluant depuis 15 jours, associée à une impotence fonctionnelle partielle du membre inférieur gauche, sans troubles vésico-sphinctériens. A l'examen initial, on a noté un niveau sensitif T4, une parésie des releveurs du pied gauche, des réflexes ostéo-tendineux vifs du côté gauche. Le bilan biologique a montré un syndrome inflammatoire biologique avec un VS accélérée à 80 mm/1ère h, et une CRP à 67 mg/l, sans perturbation du bilan phosphocalcique, ni de l'IEPP. Les explorations morphologiques (TDM + IRM) ont objectivé un tassement pathologique de D3, une ostéolyse de l'hémi-arc postérieur gauche associé à une épidurite et une infiltration tumorale intra-médullaire responsable d'une compression avec latéro-déviation de la moelle dorsale en regard (Figure 3, Figure 4 et Figure 5). D'où le patient a été opéré en urgence : il lui a été fait une laminectomie et une stabilisation du rachis dorsal, par voie postérieure (Figure 6), associée à une biopsie osseuse. L'examen histologique de la pièce opératoire avait conclu à un plasmocytome vertébral de phénotype IgG Kappa. Les suites opératoires ont été marquée par la survenue d'une embolie pulmonaire ayant bien évolué sous traitement médical. Le patient a reçu une radiothérapie à une dose de 44 Gy étalée sur 5 semaines. L'évolution s'est faite vers la régression de la tumeur sans aucun signe de transformation myélomateuse (au recul de 60 mois), avec des séquelles à type d'oesophagite post radique, et de vessie neurologique.

**Figure 7:**
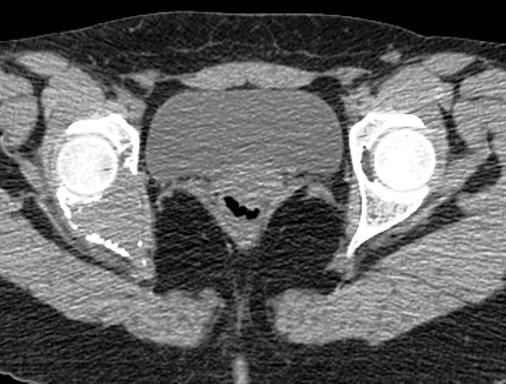
Coupe Horizontale TDM du bassin montrant un processus ostéolytique du cotyle droit, de densité tissulaire, étendue aux colonnes antérieure et postérieure, avec effraction des corticales osseuses et envahissement des parties molles en regard

**Observation 3 :** patiente âgée de 46 ans se plaint de douleurs fessière et inguinale droites d'allure mixtes évoluant depuis 4 mois, associée à une impotence fonctionnelle partielle et une boiterie du membre inférieur droit. Le bilan biologique a révélé une gammapathie monoclonale à chaînes légères lambda. Le bilan radiologique a objectivé la présence de lésions ostéolytiques au niveau du toit du cotyle droit étendue à ses colonnes antérieure et postérieure, sans remaniement de l'articulation coxo-fémorale homolatérale (Figure 7). Il lui a été fait une biopsie osseuse scanno-guidée, dont l'examen anatomopathologique a conclu à un plasmocytome solitaire du cotyle. La patiente a reçu une radiothérapie à la dose de 40 Gy, avec une évolution vers la régression de la tumeur. Au dernier recul de 24 mois, la patiente a gardé une boiterie du membre inférieur, avec usage d'une seule canne à la marche, sans aucun signe radio-clinique de récidive loco-régionale.

## Discussion

Les hémopathies plasmocytaires, dont le plasmocytome solitaire (PS) constitue moins de 10 %, représentent 1 à 2 % de l'ensemble des néoplasies humaines [2, 6–8]. Il existe deux formes cliniques de PS: le plasmocytome osseux ou intra médullaire, et le plasmocytome extra médullaire. Les formes osseuses des plasmocytomes solitaires sont les plus fréquentes [2, 3, 5, 6]. Les critères qui permettent de retenir le diagnostic de plasmocytomes solitaires sont: - une preuve histologique de l'unicité de l'atteinte - négativité des radiographies du squelette - l'absence de signes d'envahissement de la moelle osseuse [6]. Le plasmocytome solitaire osseux touche préférentiellement les hommes au cours de leur 5ème ou 6ème décennie. L'âge au moment du diagnostic est approximativement de 10 ans de moins que celui du myélome multiple [2, 5, 6]. Dans notre série, l'âge moyen de nos patients est de 43 ans (extrêmes 37-47 ans). Le PSO affecte souvent le squelette axial, surtout le rachis et le crâne. Ses circonstances de découverte sont dominées par la douleur osseuse, les signes neurologiques et la fracture pathologique [2, 6]. Le diagnostic repose sur l'examen physique, la numération sanguine, la biopsie ostéo-médullaire, l'électrophorèse des protéines plasmatiques (EPP), la recherche de protéinurie de Bence Jones, et des radiographies du squelette [5, 6]. Une gammapathie monoclonale est retrouvée dans près de la moitié des cas de plasmocytomes solitaires [6]. L'imagerie par résonance magnétique (IRM) est très performante pour l'évaluation de l'envahissement médullaire, et de l'extension vers les parties molles. Elle montre des lésions osseuses en iso-signal T1, et en hypersignal T2 qui se réhaussent après injection de produit de contraste [5–8]. Le traitement de référence des PS fait appel à la radiothérapie (RT) à des doses modérées comprises entre 40 et 50 Gy, avec taux de contrôle local satisfaisant qui varie entre 70 et 100% [1–8] (Tableau 1). L'irradiation doit concerner l'ensemble de la masse tumorale avec des marges de sécurité d'au moins 2 centimètres de tissu sain. Dans les localisations vertébrales, le volume cible doit intéresser un ou deux étages sains au-dessus, et au-dessous du niveau de la lésion [5–7]. Dans notre série, l'irradiation s'est étendue à deux étages de part et d'autre de la lésion : irradiation de D1 à D5 pour un plasmocytome de D3, et une irradiation de T11 à L3 pour un plasmocytome de L1. L'irradiation prophylactique des aires ganglionnaires satellites n'est pas toujours nécessaire, et ses indications demeurent encore controversées [3, 5, 6]. Certains auteurs ont mis en évidence l'existence d'un effet dose-dépendant de la radiothérapie, en démontrant une augmentation statistiquement significative du taux de contrôle local et du taux de survie à 5 ans par l'augmentation de la dose de radiothérapie (au-delà de 40 Gy) [1, 3, 5–7]. Le traitement est chirurgical peut être indiquée à visée diagnostique, en cas de complication neurologique telle que la compression médullaire, ou afin de traiter ou de prévenir une fracture pathologique sur un os fragilisé par l'ostéolyse tumorale. La résection chirurgicale complète peut être indiquée dans les localisations périphériques accessibles, mais elle ne doit pas être mutilante, vu l'efficacité équivalente à la radiothérapie qui 'contrairement à la chirurgie radicale- permet de préserver une bonne fonction [1, 2, 4, 6]. Dans notre série, la chirurgie a été indiquée chez un seul patient : il lui a été fait une laminectomie décompressive et une ostéosynthèse par voie postérieure. Les modalités évolutives des PS sont représentées par la récidive locale, l'envahissement ganglionnaire et la transformation myélomateuse. Le délai moyen de survenue de la récidive est de 2 ans, et toutes les récidives précédemment décrites surviennent durant les cinq premières années qui suivent le traitement [6]. Les principaux facteurs prédictifs de récidive sont la taille tumorale et la dose de la radiothérapie délivrée, avec un risque plus élevé pour les tumeurs dont la taille dépasse 5cm et en cas de radiothérapie à une dose inférieure ou égale à 35 Gy [1–3, 6]. Pour la transformation myélomateuse, les facteurs prédictifs les plus rapportés dans la littérature sont : - l'âge > 63 ans - la persistance d'une gammapthie monoclonale après la radiothérapie - la variété intra-médullaire - La localisation vertébrale - la dose de la radiothérapie - la taille tumorale > 5 cm [1, 2, 5–7]. Dans l'étude que nous rapportons, les facteurs de mauvais pronostic que nous avons notés sont le siège vertébral pour 2 patients et la variété intra-médullaire pour tous les patients. Aucun patient n'avait présenté une gammapthie monoclonale, et aucun n'a évolué vers la récidive locorégionale ni vers la dissémination vu le recul moyen relativement faible (32 mois). Le facteur le plus couramment retenu dans la littérature est la persistance de la gammapathie monoclonale, une année après la fin de la dernière cure de radiothérapie [1, 2; 5–7]. Elle justifierait, pour certains auteurs, la prescription d'une chimiothérapie (CT) adjuvante préventive, dont l'efficacité reste à démontrer [1, 2, 5, 6].

**Tableau 1:** Revue de la littérature

Série	Nombre	Recul	Localisation	Traitement	Evolution
Kochbati (2004)	13	63 mois	-Rachis (6 cas) -os plats (6 cas) -os longs (1 cas)	-RT pour tous les patients -Chirurgie (8 cas) -CT (3 cas)	-récidive locale (2 cas) -MM (4 cas)
Moukhlissi (2011)	10	-	-Rachis (6 cas) -bassin (2 cas) -crâne (2 cas)	-RT pour tous les patients -Chirurgie (4 cas)	-déficit neurologique séquellaire (2 cas)
BenCheikh (2006)	1	13 mois	-Mandibule	-Chirurgie + RT	-Favorable
Bousnina (2006)	1	12 mois	-Côtes	-RT	-Favorable
Liebross (1998)	57	-	-Rachis (40 cas) -Bassin (17 cas) -Côtes (14 cas) -Omoplate (9 cas) -Sternum (7 cas) -Crâne (5 cas) -Autres localisations (8 cas)	-RT (56 cas) -Chirurgie seule (1cas)	-MM (29 cas) -Récidive locale (2 cas)
**Notre série**	3	32 mois	-Rachis (2 cas) -Bassin (1 cas)	-RT pour tous les patients -Chirurgie (1 cas)	-déficit neurologique séquellaire (1 cas)

## Conclusion

Le plasmocytome solitaire osseux est une tumeur maligne rare qui atteint souvent le squelette axial. La plupart des patients sont traitées par radiothérapie seule à doses modérées, d'autres nécessitent une intervention chirurgicale. La chimiothérapie adjuvante n'a aucune place. Le taux de contrôle local est souvent satisfaisant. La complication la plus redoutable est la survenue d'un myélome multiple (MM) qui est plus fréquente, comparativement aux formes extra médullaires. Une meilleure prise en charge est tributaire de progrès dans le traitement du myélome multiple chez les patients présentant des signes de dissémination systémiques.
